# Mesenteric Artery Thrombosis, Microvascular Intestinal Endothelitiis, and Guillain-Barrè Syndrome in the Same SARS-CoV-2 Patient

**DOI:** 10.7759/cureus.11326

**Published:** 2020-11-04

**Authors:** Luciano E Ferraris, Giuseppe Sala, Stefano Casalino, Luigi Losurdo, Valentino De Filippis

**Affiliations:** 1 Anaesthesia and Intensive Care, Istituto Clinico Città Studi (ICCS), Milan, ITA; 2 Anaesthesia and Intensive Care, Military Hospital, Milan, ITA

**Keywords:** mesenteric artery thrombosis, guillain barrè syndrome, sars-covid2, predictors

## Abstract

Coronavirus disease 2019 (COVID-19) in humans is a novel disease that can affect every organ of the body, with life-threatening consequences. Microvascular lesions and thrombosis have been previously reported in the lung, kidney, and brain. We report a case of combined intestinal lesions and Guillain-Barrè Syndrome in a patient suffering from COVID-19 in the absence of clear laboratory predictors of upcoming complications. The patient survived the severe respiratory syndrome but died after virus-related systemic organ failure.

## Introduction

Like other common viruses, coronavirus disease 2019 (COVID-19) may affect every organ and apparatus after primary impairment of the lungs that may lead to life-threatening conditions. The dysregulation of the inflammatory response has been speculated on to explain most of the severe complications. Laboratory findings, such as lupus anticoagulant (LAC), antiphospholipid antibodies, and low antithrombin III serum levels, have been recently reported in association with COVID-19 patients. Despite these preliminary works, we don't have clear predictors of the action of the virus on the body. Drugs used for arthritis therapy, such as tocilizumab, were tested with poor and unclear results and, therefore, abandoned. In this report, we describe two major complications - mesenteric artery thrombosis and Guillain-Barrè Syndrome - in a patient with a favorable evolution of bilateral interstitial pneumonia.

## Case presentation

**Figure 1:**
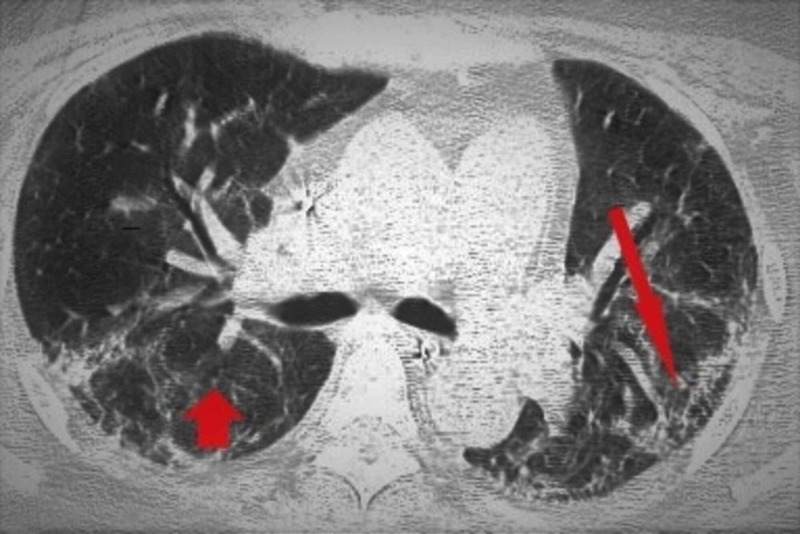
Thorax CT scan: bilateral interstitial pneumonia (arrows)

A 65-year-old Hispanic woman, height 160 cm, and weight 75 kg, with a past medical history of depression and fibromyalgia, was admitted in the COVID-19 medical sub-intensive ward and subsequently transferred to the intensive care unit for worsening respiratory symptoms. Computed tomography (CT) demonstrated bilateral interstitial pneumonia (Figure 1).

**Figure 2:**
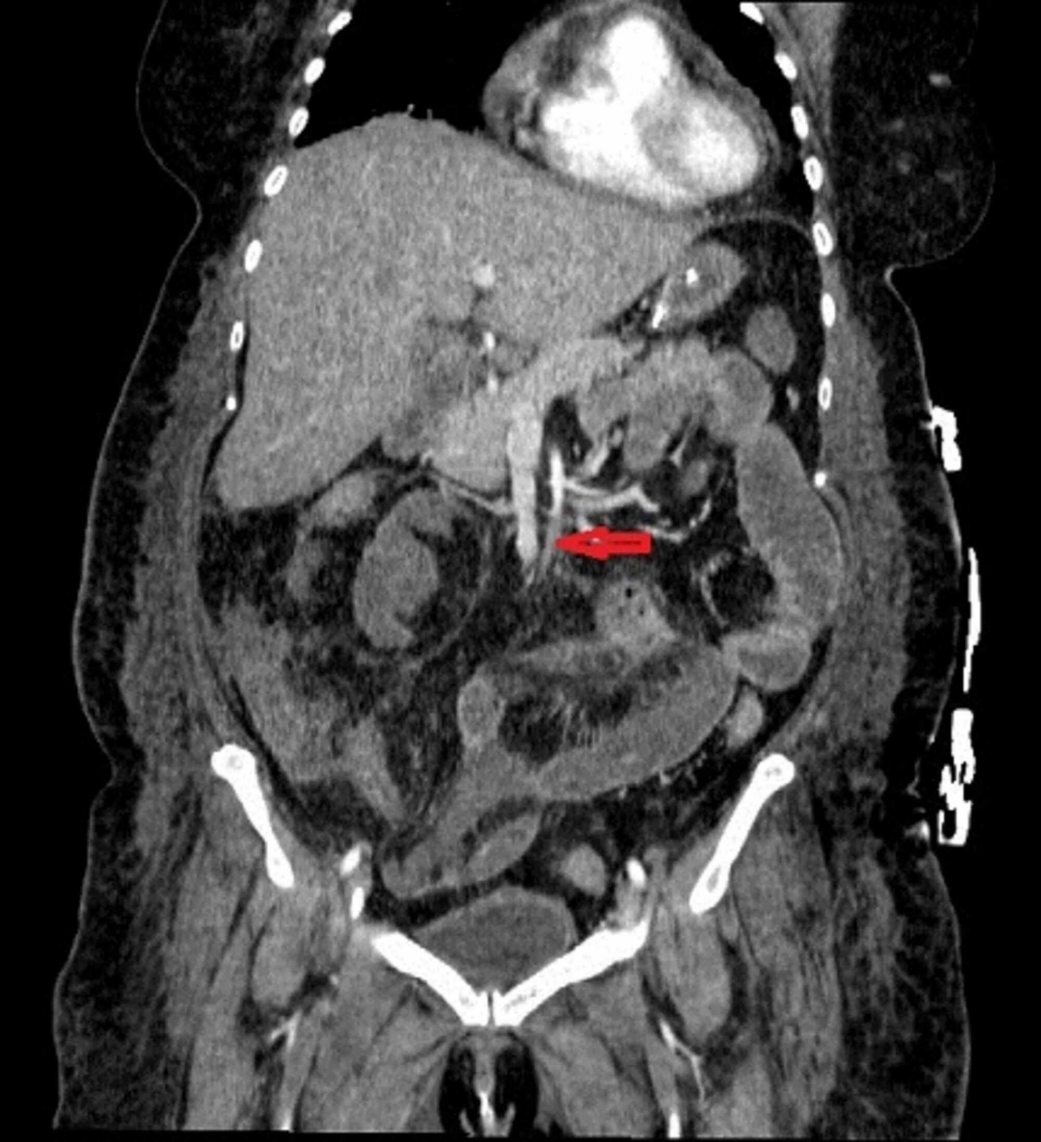
CT scan coronal view: upper mesenteric artery thrombosis (arrow)

**Figure 3:**
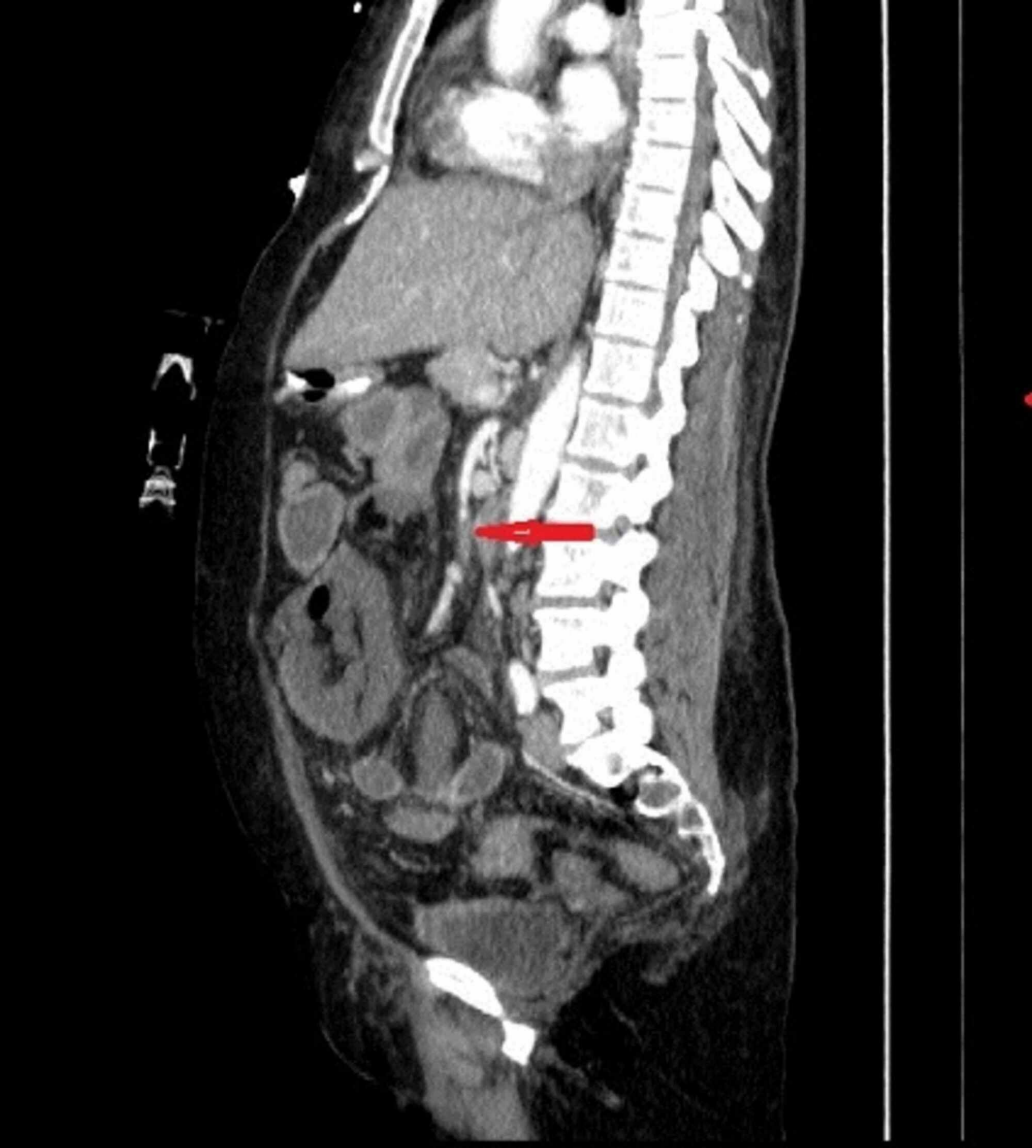
CT scan sagittal view: upper mesenteric artery thrombosis (arrow)

On arrival, she was intubated and ventilated as partial pressure of oxygen (PaO2)/fraction of inspired oxygen (FiO2) (P/F) = 71 (n.r. > 400), FiO2=1 in volume-controlled mandatory ventilation (CMV). Ventilation was set according to lung-protective ventilation recommendations and up-to-date guidelines [1] to reduce the associated risk of ventilatory-induced lung injury (VILI): tidal volume (TV) 6 ml/kg of ideal body weight (IBW), and plateau pressure <29 cm H_2_O. Respiratory rate (RR) was maintained between 18-22 breaths per minute and positive end-expiratory pressure (PEEP) was kept under the level of 12 cm H_2_O. The prone position gave a significant rise in the PaO2 value by 25% and was adopted for 12-14 hours daily [2]. We accepted pulse oximetry (SpO2) values of 90%-92%. Sedation and muscle relaxation were obtained by the continuous infusion of propofol, remifentanil, and rocuronium with target bispectral index (BIS) values and train of four (TOF) target 40-60 and 25%, respectively. She was febrile (39.5°C), with clinical signs of septic shock, and was treated with blood volume replacement, norepinephrine, and epinephrine. Laboratory tests revealed white blood cells (WBC) 47.5 10^3^/ml (n.r. 4-10 10^3^/ml), C-reactive protein (CRP) 33.9 mg/L (n.r. < 6 mg/L), procalcitonin (PCT) 4.69 ng/ml (n.r. < 0,5 ng/ml), D-dimer 8,255 ng/ml (n.r. < 500 ng/ml), lymphocyte 2,500/µL (n.r. 1,000 - 4,800/µL), coagulation tests in normal range, and normal platelet (PLT) count. The pharmacological therapy was: enoxaparine 80 mg/day, reduced to 40 mg/day when renal insufficiency occurred, hydroxychloroquine 400 mg/day for five days, azithromycin 0.5 g for five days, tocilizumab 8 mg/kg once daily for two days, lopinavir/ritonavir 400 mg/day, methylprednisolone 20 mg/day for three days. Antithrombin (AT) serum levels were strictly monitored and even corrected if under 80%. Antibiotics such as meropenem, tigecycline, vancomycin, colistin, ciprofloxacin, and anidulafungin were given for intercurrent bacterial and fungal infections revealed by bronchoalveolar lavage (BAL) and blood and urine culture. In the first septic shock, Staphylococcus aureus was isolated in the blood samples. Four days after admission to the intensive care unit (ICU), she developed acute kidney injury (AKI) and was consequently treated with continuous venovenous hemofiltration (CVVH) that lasted for seven days. Notwithstanding these still critical conditions, blood gas exchange had a favorable trend (P/F 220, 293, 320) and pressure support ventilation (PSV) was well-tolerated after percutaneous Griggs tracheotomy was still possible with acceptable laboratory coagulation values. On the 13th day, she had acute abdominal distension and worsening cardiovascular conditions, CT of the abdomen documented a filling defect of the upper mesenteric artery (Figures 2-3).

**Figure 4:**
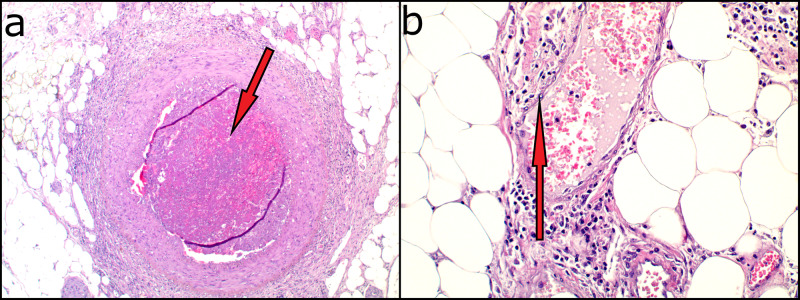
Histopathology report a: Massive thrombosis occluding a perivisceral arteriola. H&E, 50 x b: Subtle modifications of endothelial cells with enlarged, reactive nuclei and abundant granular cytoplasm. H&E, 200 x H&E: hematoxylin and eosin

The patient underwent laparotomy and resection of the terminal ileus plus right hemicolectomy. The immediate postoperative period was complicated by intraperitoneal bleeding and then operated. On re-exploration, the surgeons found many points of active bleeding without intestinal wall disruption. A histopathological examination of the surgical sample, right colon, and terminal ileus highlighted massive thrombosis of the intestinal vessels and signs of vasculitis and endotheliitis (Figure 4) [3].

**Figure 5:**
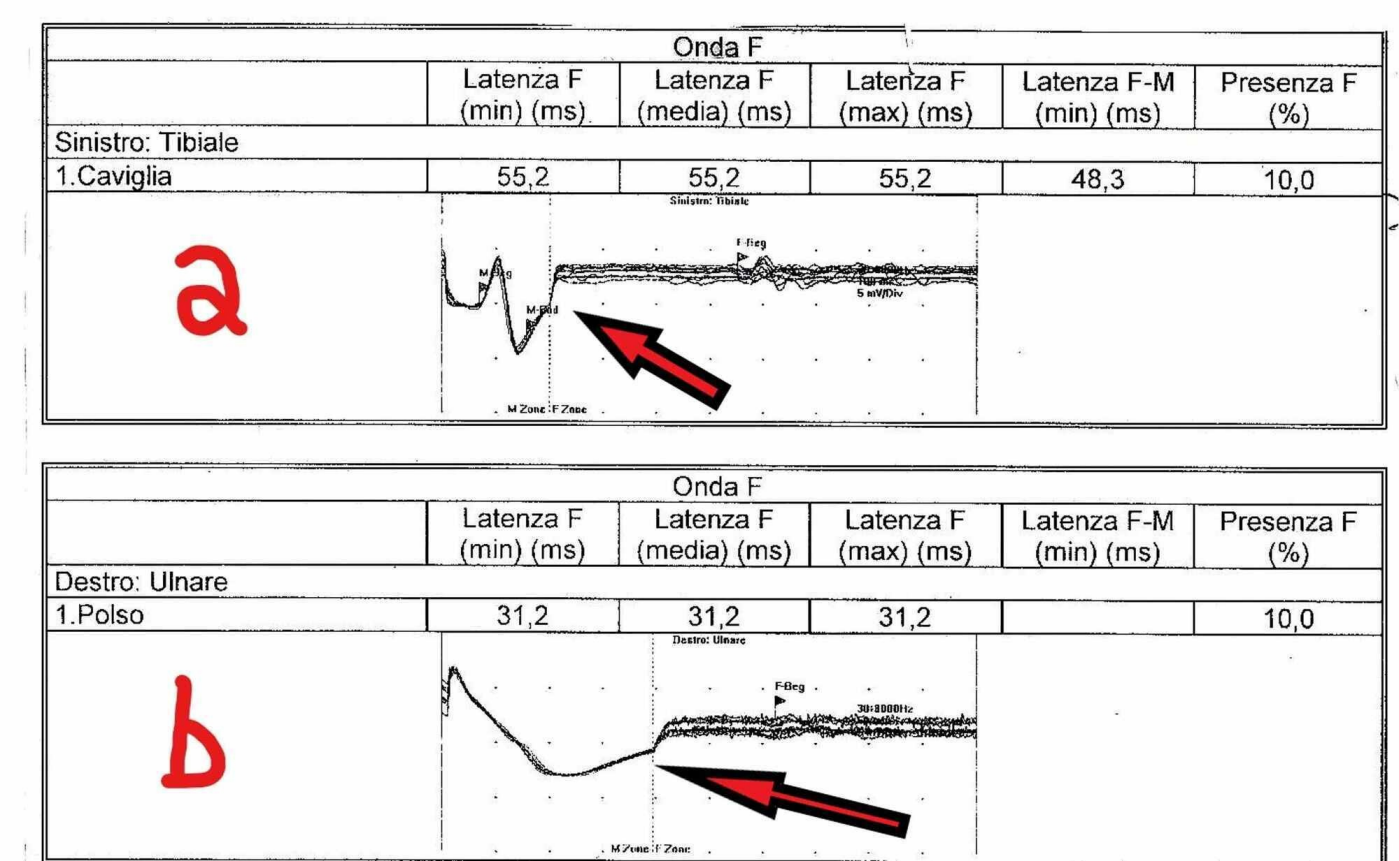
EMG with prolonged F waves (arrows) a: Left tibial nerve; b: Right ulnar nerve EMG: electromyography

After five days of the associated postoperative period, she developed progressive paralysis of the four limbs; no acute alterations of the brain and brainstem were evidenced by magnetic resonance imaging (MRI). Neurological examination, cerebrospinal fluid (CSF), and electromyography (EMG) (Figure 5) confirmed the diagnosis of Guillain-Barrè syndrome, which was treated with immunoglobulin G (IgG) 30 g/day for seven days.

Laboratory tests revealed an increase in interleukin 6 (IL6) up to 230 pg/ml (n.r. ≤ 1.8 pg/ml) that slowly decreased to 4.5 pg/ml 12 days later. Despite unchanged low molecular weight heparin (LMWH) therapy, we observed a prolonged partial thromboplastin time (PTT) of up to 63.1 sec (n.r. 30 - 40 sec.) without other abnormal values of blood coagulation. Other markers, such as complement C3, C4, lupus anticoagulant (LAC), antinuclear antibodies (ANA), and anti-cytoplasmic antibodies (ANCA), were absent or in the normal range [4]. The therapy was useful with the near-complete restoration of neuromuscular conditions, she also began a physiokinesitherapy program with satisfying compliance. Mild but continuous bleeding from ileostomy was noticed and endoscopy of the stoma pointed out a terminal small artery, which was endoscopically clipped. Despite this treatment, the bleeding didn't stop. Surgeons decided for a second laparotomic look to check out intestinal and vascular conditions. The patient had a reoperation and recanalization was performed. Histopathological examination of the ileus described the diffusive and massive endotheliitis of the vessels. The postoperative was complicated by sepsis quickly evolving in multiple organ failure (MOF); the patient died after two months of hospital stay.

## Discussion

Severe COVID-19 acute respiratory insufficiency and failure is now considered different from the well-known adult respiratory distress syndrome (ARDS). In the early stages, we found a normal or minimal reduction in pulmonary compliance, and mechanical ventilation was easy to perform, ranging between safe pressure limits [5-6]. In this case, it was more difficult because of the associated bacterial infection and very low P/F, and only a positive response in the prone position led to a slow but significant improvement. This patient had a fast evolution toward typical ARDS and then was treated with good results. Laboratory investigations never demonstrated significant coagulopathy; when received, blood transfusion, plasma, and low antithrombin (AT) serum levels were compatible with consumption, and tests never met diffusive intravascular coagulation (DIC) criteria or other thrombocytopenic disorders [7]. The acute thrombosis of the upper mesenteric artery showed up when pulmonary conditions were improving and inflammatory indices were under control.

Endothelial cell infection has been recently described in COVID-19 patients who died of multiple organ failure (MOF) by electron microscopy of the lung, kidney, and ileum [8]. The inflammatory response in COVID-19, despite important multiorgan damage, is not a cytokine storm-like disease such as typical ARDS [9]. According to this report, we found a mild or moderate increase in IL6 with a rising at the onset of Guillain-Barrè syndrome. Other markers, such as antiphospholipid antibodies, LAC, C3, C4, ANA, and ANCA appeared normal and only IL6 was high - around 230 pg/ml. The early bacterial infection and sepsis may have switched the pulmonary conditions toward the phenotype more consistent with ARDS and fast improvement in blood gas exchange by prone position. The histopathologic examination of the colon and ileum described thrombosis of the vessels and endotheliitis well. The Guillain-Barrè syndrome with typical albuminocytologic dissociation and absent virus in CSF was not different from the well-known disease based on immune-mediated disorder. In this case, the dysregulated inflammation seems to be linked to immunosuppression and endothelial damage rather than a hyperinflammatory cascade. Recent publications reported a higher level of serum LAC or antiphospholipid antibodies in COVID-19 patients [10-11] as compared to our case. We can speculate that damage-associated molecular patterns (DAMPs) triggered by the virus might explain the diffusive vascular endotheliitis in this particular clinical case.

## Conclusions

This case demonstrates that COVID-19, apart from severe respiratory involvement in the early stages, may be much more harmful and progress to a serious and unpredictable systemic disease despite the resolution of pulmonary symptoms. Despite referred and published associated alterations of inflammatory laboratory findings, we have not reported a significant elevation of the serum inflammatory markers hypothesized as predictors of the extrapulmonary manifestations of COVID-19.
